# Physiological and biochemical mechanisms associated with trehalose-induced copper-stress tolerance in rice

**DOI:** 10.1038/srep11433

**Published:** 2015-06-15

**Authors:** Mohammad Golam Mostofa, Mohammad Anwar Hossain, Masayuki Fujita, Lam-Son Phan Tran

**Affiliations:** 1 Laboratory of Plant Stress Responses, Department of Applied Biological Science, Faculty of Agriculture, Kagawa University, Miki, Kagawa 761-0795, Japan; 2 Department of Biochemistry and Molecular Biology, Bangabandhu Sheikh Mujibur Rahman Agricultural University, Gazipur 1706, Bangladesh; 3 Department of Genetics and Plant Breeding, Bangladesh Agricultural University, Mymensingh 2202, Bangladesh; 4 Signaling Pathway Research Unit, RIKEN Center for Sustainable Resource Science, 1-7-22 Suehiro-cho, Tsurumi, Yokohama 230-0045, Japan

## Abstract

In this study, we examined the possible mechanisms of trehalose (Tre) in improving copper-stress (Cu-stress) tolerance in rice seedlings. Our findings indicated that pretreatment of rice seedlings with Tre enhanced the endogenous Tre level and significantly mitigated the toxic effects of excessive Cu on photosynthesis- and plant growth-related parameters. The improved tolerance induced by Tre could be attributed to its ability to reduce Cu uptake and decrease Cu-induced oxidative damage by lowering the accumulation of reactive oxygen species (ROS) and malondialdehyde in Cu-stressed plants. Tre counteracted the Cu-induced increase in proline and glutathione content, but significantly improved ascorbic acid content and redox status. The activities of major antioxidant enzymes were largely stimulated by Tre pretreatment in rice plants exposed to excessive Cu. Additionally, increased activities of glyoxalases I and II correlated with reduced levels of methylglyoxal in Tre-pretreated Cu-stressed rice plants. These results indicate that modifying the endogenous Tre content by Tre pretreatment improved Cu tolerance in rice plants by inhibiting Cu uptake and regulating the antioxidant and glyoxalase systems, and thereby demonstrated the important role of Tre in mitigating heavy metal toxicity. Our findings provide a solid foundation for developing metal toxicity-tolerant crops by genetic engineering of Tre biosynthesis.

Environmental pollution by heavy metals has been known for a long time; however, exposure to heavy metals still continues and is worsening, particularly in less developed countries^1^,^2^,^3^. In this context, copper (Cu) has emerged as a serious pollutant in the past few decades because of its excessive use in the manufacturing and agricultural industries^4^,^5^. In Bangladesh, cultivated lands are highly contaminated with Cu because of uncontrolled and repeated application of Cu-containing pesticides, pig and poultry slurries, and untreated wastewater from industrial establishments^6^. Rice (*Oryza sativa*) is an important cereal crop grown across the world and is also considered a staple food in many Asian countries, including Bangladesh, India, China, and Japan. Bangladesh is the world’s fourth biggest producer of rice, with 11.54 million ha under cultivation^7^. Cultivation of crops, such as rice, in Cu-polluted lands, results in yield loss, reduced seed quality, and Cu toxicity in humans. Therefore, developing rice varieties tolerant to Cu stress or understanding the overall mechanism of rice plant response to Cu toxicity is important for sustainable rice production.

Cu, as a co-factor of numerous proteins and enzymes, is involved in plant growth, development, and protective mechanisms^8^. However, a slightly higher than optimal level of Cu is toxic and can detrimentally affect biochemical and physiological processes, including photosynthesis, nitrogen metabolism, senescence, membrane integrity, and mineral uptake in plants^9^,^10^. Cu toxicity also causes abnormal root morphology, chlorosis, necrosis, and rolling in leaves, all of which hinder plant growth and development, and ultimately lead to reduced crop productivity^10^,^11^,^12^,^13^. The inherent redox active nature of Cu also potentiates Cu toxicity by generating reactive oxygen species (ROS), such as superoxide anion (O_2_^•−^), hydrogen peroxide (H_2_O_2_), and hydroxyl radical (OH^•^)^12^,^14^,^15^. To combat oxidative stress, plant cells are well equipped with intrinsic antioxidant capacity that comprises enzymatic components, such as superoxide dismutase (SOD), catalase (CAT), glutathione peroxidase (GPX), glutathione *S*-transferase (GST), ascorbate peroxidase (APX), dehydroascorbate reductase (DHAR), monodehydroascorbate reductase (MDHAR), and glutathione reductase (GR), as well as non-enzymatic components, such as ascorbic acid (AsA) and glutathione (GSH)^16^.

Methylglyoxal (MG), a highly reactive compound, is known to accumulate under abiotic stresses, including heavy metal toxicity^17^,^18^. Besides its direct cytotoxic effects on biological biomolecules, MG induces oxidative stress either directly through increasing ROS formation or indirectly by forming advanced glycation end products (AGEs)^19^,^20^. To detoxify MG, plants possess a GSH-dependent glyoxalase (Gly) system that converts MG into D-lactate by employing Gly I and Gly II^21^. Transgenic plants overexpressing *Gly* genes have shown higher tolerance to toxic levels of zinc, cadmium, and lead^22^, as well as higher ROS detoxification capacity^17^,^23^. Efficient induction of the antioxidant defense and Gly systems correlates with increased tolerance to abiotic stresses^12^,^24^,^25^.

Regulating Cu homeostasis is crucial in maintaining the intracellular Cu level to avoid toxicity. Plants have developed various mechanisms to restrict Cu toxicity, such as inhibition of Cu uptake by binding with root exudates like organic acids, intracellular sequestration by strong ligands like cysteine-rich compounds and phytochelatins, and exclusion of excessive Cu from the cells by sugar alcohols like trehalose (Tre)^26^,^27^,^28^. Tre, a non-reducing disaccharide of glucose, protects plant cells against long-term desiccation by stabilizing enzymes, proteins, and biological membranes under dehydration^29^. Tre is highly compatible with cellular metabolism because it is non-reactive even at higher concentrations^29^. Tre has the added advantage of being a signaling and antioxidant molecule and it can mitigate several types of abiotic stress, including heat, drought, and salinity^30^,^31^,^32^,^33^,^34^. However, the beneficial role of Tre and the associated mechanisms involved in protecting plants against heavy metal toxicity remain elusive.

Thus, in the current study, we examined the effects of excessive Cu on growth and development of the economically important rice crop, as well as various physiological and biochemical parameters in the plants. More important, to gain an insight into the physiological and biochemical mechanisms Tre uses to enhance tolerance in rice plants to Cu stress, we investigated the effects of exogenous Tre on (i) Cu uptake and accumulation, (ii) Cu-induced changes in growth parameters and the levels of oxidative parameters, (iii) Cu-induced modulation of non-enzymatic antioxidants, and (iv) the activities of the enzymes involved in the antioxidant defense and Gly systems in rice seedlings under Cu stress.

## Results

### Plant growth parameters

Several plant growth parameters, including plant height, fresh weight (FW), and dry weight (DW), were determined to estimate the negative effects of excessive Cu on plant growth and the potential mitigation effects of Tre on Cu-stressed rice seedlings. The height of the Cu-stressed seedlings decreased by 13 and 18% at days 4 and 7, respectively, compared with control (Table 1). However, under Cu stress, Tre pretreatment resulted in a significant increase in plant height, almost reaching that of control, compared with Cu-stressed only seedlings. FW of the Cu-stressed seedlings decreased by 33 and 65%, whereas DW decreased by 17 and 39% at days 4 and 7, respectively, compared with control. However, FW and DW remained significantly higher at both days 4 and 7 in the Tre-pretreated Cu-stressed seedlings relative to the Cu-stressed only seedlings. Tre pretreatment alone did not markedly affect plant height, FW, and DW compared with control over the experimental period. These results demonstrated that Tre pretreatment could enhance tolerance of rice seedlings under Cu stress.

### Relative water content (RWC), and chlorophyll (Chl), and proline (Pro) content

Under Cu stress, RWC and total Chl content decreased significantly at days 4 and 7 compared with control (Table 1). The decreases were notably drastic (32 and 61% for RWC and Chl content, respectively) after 7 days of Cu treatment. In contrast, a lower decrease in RWC (5 and 20% at days 4 and 7, respectively) and total Chl content (3 and 44% at days 4 and 7, respectively) was observed in the Tre-pretreated Cu-stressed seedlings compared with control. However, RWC and total Chl content remained significantly higher in the Tre-pretreated Cu-stressed seedlings compared with the Cu-stressed only seedlings, suggesting that Tre could alleviate the negative effects of excessive Cu on the RWC and Chl content of rice plants. Under non-stressed conditions, no significant changes in RWC and total Chl content were observed in the Tre-pretreated seedlings relative to control. The level of Pro, which plays an osmoprotectant role against osmotic disturbance in plant cells caused by various abiotic stresses, including heavy metal stress^35^,^36^, increased by 489 and 566% at days 4 and 7, respectively, in the Cu-stressed seedlings compared with control (Table 1). On the other hand, compared with the Cu-stressed rice plants, the Tre-pretreated Cu-stressed seedlings showed a lower increase in Pro content (218 and 217%, respectively) over that of control. No significant change in Pro content was noted upon Tre pretreatment under non-stressed conditions over the experimental period.

### Cu content in roots and leaves

**Figure 1:**
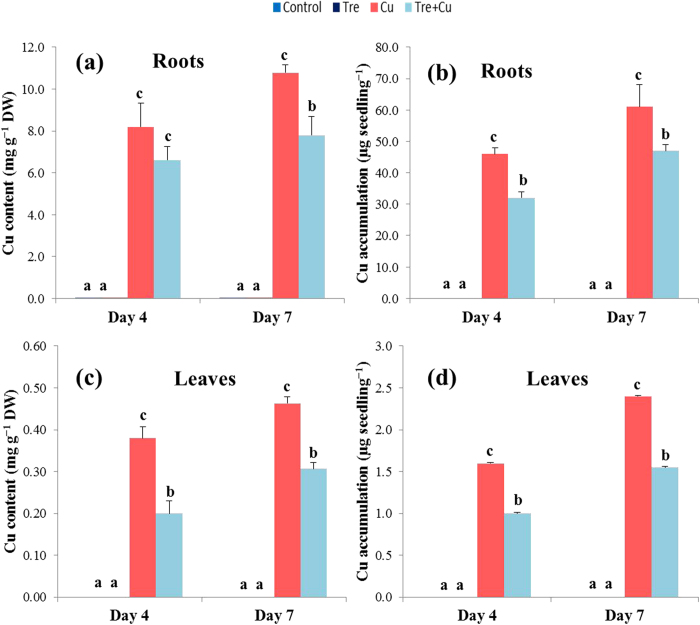
Effect of exogenous trehalose on Cu content and accumulation in rice seedlings with or without Cu stress. (**a**) Cu content in roots, (**b**) Cu accumulation in roots, (**c**) Cu content in leaves, and (**d**) Cu accumulation in leaves. Control, Tre, Cu, and Tre+Cu correspond to control, 10 mM trehalose, 100 μM CuSO_4_, and 10 mM trehalose + 100 μM CuSO_4_, respectively. Bars represent standard deviation (SD) of the mean (*n* = 3). Different letters indicate significant differences among treatments at *p* < 0.05, according to Duncan’s multiple range test.

To examine whether the positive impact of Tre pretreatment on Cu-stressed seedlings was associated with its ability to reduce Cu uptake, the Cu content and accumulation were determined in the roots and leaves of the rice seedlings with or without treatment with Cu stress. Cu content in the roots of the Cu-stressed seedlings was 24 and 38% higher at days 4 and 7, respectively, compared with the Tre-pretreated Cu-stressed seedlings (Fig. 1a). The accumulation of Cu decreased by 30 and 23% at days 4 and 7, respectively, in the Tre-pretreated Cu-stressed seedlings compared with those in the seedlings treated with Cu stress alone (Fig. 1b). Similarly, Cu content in the leaves of Cu-stressed seedlings was 90 and 48% higher, but Cu accumulation decreased by 38 and 35% at days 4 and 7, respectively, compared with the Cu-stressed only seedlings (Fig. 1c,d)

### Malondialdehyde (MDA) and H_2_O_2_ content and lipoxygenase (LOX) activity

H_2_O_2_ and MDA content were measured as the index of oxidative stress. Both these oxidative parameters significantly increased in the Cu-stressed seedlings over control at both days 4 and 7 (Table 2), but the increases were more drastic (151 and 119% for H_2_O_2_ and MDA, respectively) at day 7 of Cu treatment. On the other hand, the levels of H_2_O_2_ and MDA in the Cu-stressed seedlings pretreated with Tre were 5 and 17% lower at day 4, and 43 and 30% lower at day 7, respectively, compared with those in the Cu-stressed only seedlings, indicating that Tre could protect rice plants against excessive Cu-induced oxidative stress. Under non-stressed conditions, H_2_O_2_ content decreased by 11% at day 4 in the Tre-pretreated seedlings compared with the untreated control; however, no change in MDA content was observed at both days 4 and 7. LOX, as an oxidative enzyme, can also initiate lipid peroxidation, leading to the formation of MDA^37^. In the Cu-stressed seedlings, LOX activity increased by 63 and 61% at days 4 and 7, respectively, compared with control. However, LOX activity was 12 and 24% lower at days 4 and 7, respectively, in the Cu-stressed seedlings pretreated with Tre compared with the Cu-stressed only seedlings. Under non-stressed conditions, a significant change in LOX activity was recorded after only 4 days of Tre pretreatment of rice seedlings, with an increase of 26% in the Tre-pretreated seedlings relative to control.

### ROS (O_2_^•–^ and H_2_O_2_) accumulation

**Figure 2:**
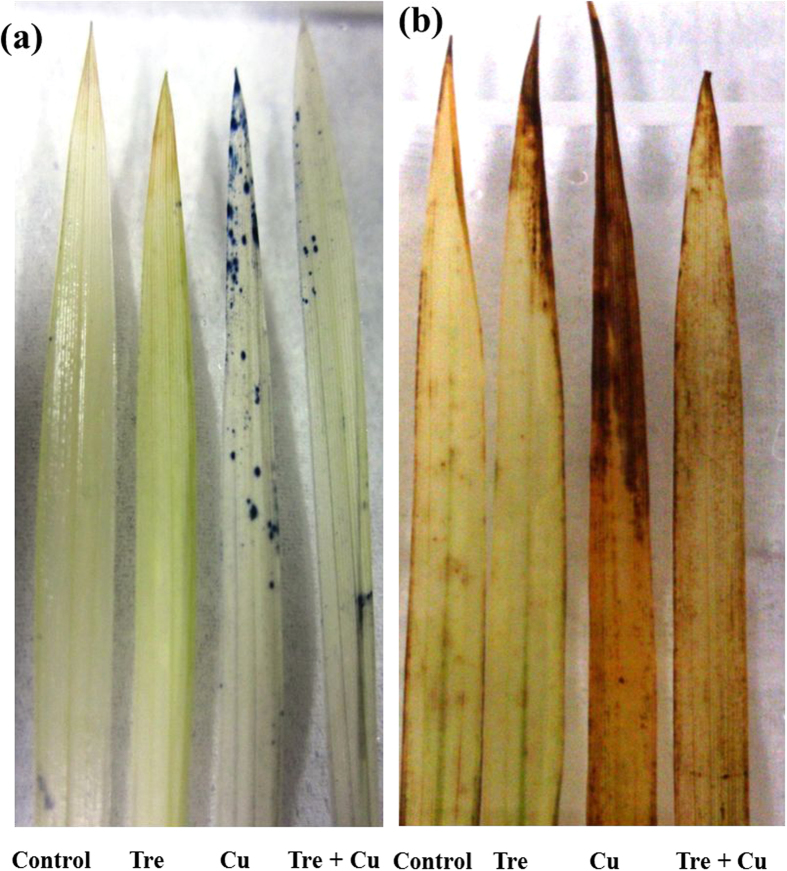
Effect of exogenous trehalose on ROS accumulation in leaves of rice seedlings with or without Cu stress. (**a**) superoxide (O_2_^∙−^) and (**b**) hydrogen peroxide (H_2_O_2_) production in rice leaves were detected using nitroblue tetrazolium (NBT) solution and 3,3'-diaminobenzidine (DAB), respectively, at day 7 of Cu stress. Control, Tre, Cu, and Tre+Cu correspond to control, 10 mM trehalose, 100 μM CuSO_4_, and 10 mM trehalose + 100 μM CuSO_4_, respectively.

ROS level is also a good indicator of oxidative stress in plants. The accumulation of O_2_^•−^ and H_2_O_2_ in the second leaf of the rice seedlings was recorded at day 7 of Cu treatment (Fig. 2). NBT staining indicated an increased amount of O_2_^•–^ as scattered dark blue spots in the leaf plate of the Cu-stressed seedlings compared with the non-treated control (Fig. 2a). Similarly, DAB staining confirmed a marked increase in brown polymerization products, which indicated the over-accumulation of H_2_O_2_ in the leaves of the Cu-stressed seedlings relative to control (Fig. 2b). More important, the accumulation of O_2_^•–^ and H_2_O_2_ in the leaves of the Tre-pretreated Cu-stressed seedlings diminished considerably compared with those of the Cu-stressed only seedlings. Pretreatment with exogenous Tre might therefore enhance tolerance in rice plants against oxidative stress induced by excessive Cu.

### Ascorbic acid (AsA) content and AsA to dehydroascorbic acid (DHA) ratio (AsA/DHA)

Compared with the untreated control, total AsA content declined significantly at both days 4 and 7 in the Cu-stressed seedlings, with a more drastic decrease (54%) observed at day 7 (Table 3). On the other hand, total AsA content in the Tre-pretreated Cu-stressed seedlings was 39 and 93% higher at days 4 and 7, respectively, compared with the Cu-stressed only seedlings. Total AsA content in the Tre-pretreated Cu-stressed seedlings was 15% higher at day 7 than at day 4. Tre pretreatment significantly increased the levels of total AsA in the rice seedlings compared with control during the experiment, under normal conditions. The AsA/DHA ratio decreased significantly in the Cu-stressed seedlings at both days 4 and 7 relative to control, and this decrease was more severe (241%) at day 7. Although the AsA/DHA ratio in the Tre-pretreated Cu-stressed seedlings was lower than that of control, the values were still significantly higher than those of the Cu-stressed seedlings at both days 4 and 7.

### GSH content and GSH to oxidized GSH (GSSG) ratio (GSH/GSSG)

In the Cu-stressed seedlings, the GSH level increased by 66 and 73% at days 4 and 7, respectively, over control (Table 3). On the other hand, Tre pretreatment restricted the increase in the GSH level in the Tre-pretreated Cu-stressed seedlings to 6 and 36% at days 4 and 7, respectively, compared with the Cu-stressed only seedlings. However, the GSH levels were still significantly higher (56 and 10% at days 4 and 7, respectively) in the Tre-pretreated Cu-stressed seedlings relative to the non-treated control. No significant change in the level of GSH was noted upon Tre pretreatment only over non-treatment at both days 4 and 7. The GSH/GSSG ratio showed a significant decrease in Cu-stressed seedlings compared with control at both time points. However, GSH/GSSG ratios were significantly higher in the Tre-pretreated Cu-stressed seedlings than those of the Cu-stressed only seedlings at both days 4 and 7.

### Antioxidant enzymes SOD, CAT, GPX, and GST

**Figure 3:**
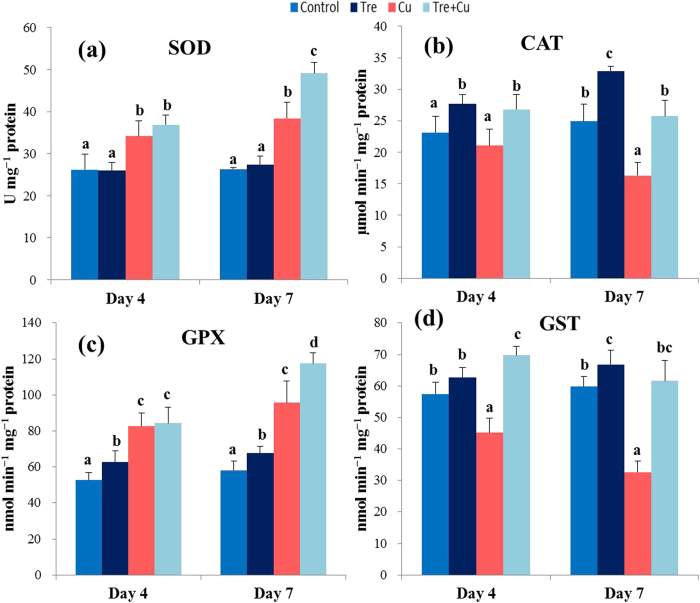
Effect of exogenous trehalose on the activities of antioxidant enzymes in rice seedlings with or without Cu stress. (**a**) superoxide dismutase (SOD), (**b**) catalase (CAT), (**c**) glutathione peroxidase (GPX), and (**d**) glutathione reductase (GST). Control, Tre, Cu, and Tre+Cu correspond to control, 10 mM trehalose, 100 μM CuSO_4_, and 10 mM trehalose + 100 μM CuSO_4_, respectively. Bars represent standard deviation (SD) of the mean (*n* = 3). Different letters indicate significant differences among treatments at *p* < 0.05, according to Duncan’s multiple range test.

Tre pretreatment affected the activities of the enzymes in a time-dependent manner. SOD activity increased by 31 and 46% at days 4 and 7, respectively, in the Cu-stressed seedlings relative to control (Fig. 3a). SOD activity was observed to further increase in the Cu-stressed seedlings subjected to Tre pretreatment, and compared with the Cu-stressed seedlings alone, its activity increased by 8 and 28% at days 4 and 7, respectively. Tre pretreatment did not alter SOD activity under non-stressed conditions compared with control over the experimental period. As shown in Fig. 3b, CAT activity remained unchanged at day 4 but drastically decreased (34%) at day 7 in the Cu-stressed seedlings compared with control. However, CAT activity in the Tre-pretreated Cu-stressed seedlings remained at the level of control at both days 4 and 7. CAT activity increased by 19 and 32% at day 4 and day 7, respectively, in the Tre-pretreated only seedlings compared with control. GPX activity increased by 56 and 65% at days 4 and 7, respectively, in the Cu-stressed seedlings relative to control (Fig. 3c). On the other hand, an increase in GPX activity was also observed (2 and 23% at days 4 and 7, respectively) in the Tre-pretreated Cu-stressed seedlings compared with the Cu-stressed only seedlings. Under non-stressed conditions, GPX activity in the Tre-pretreated seedlings increased by 19 and 16% at days 4 and 7, respectively, compared with control. GST activity, upon Cu exposure, drastically decreased by 31 and 45% at days 4 and 7, respectively, compared with control (Fig. 3d). However, an increase in GST activity (54 and 89% at day 4 and day 7) was recorded in the Tre-pretreated seedlings compared with the Cu-stressed only seedlings. A significant increase in GST activity was also recorded in the Tre-pretreated seedlings, but only at day 7, relative to control.

### Ascorbate-glutathione cycle enzymes APX, MDHAR, DHAR, and GR

**Figure 4:**
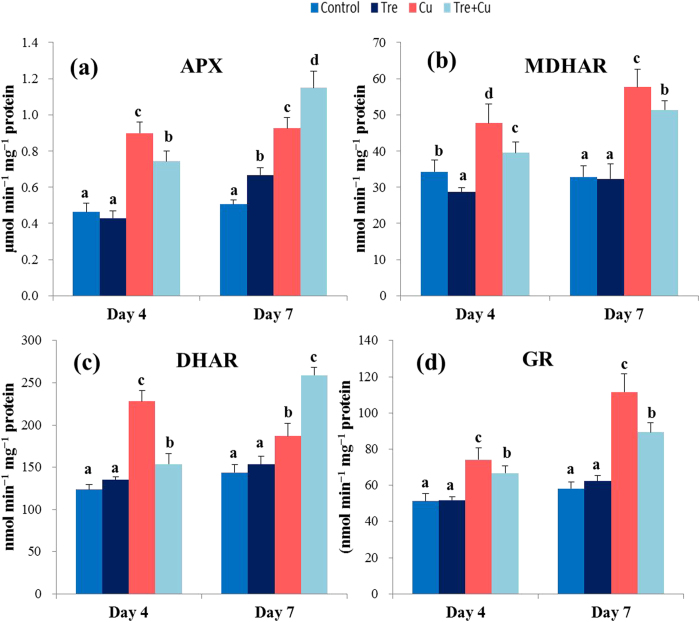
Effect of exogenous trehalose on activity of ascorbate-glutathione cycle enzymes in rice seedlings with or without Cu stress. (**a**) ascorbate peroxidase (APX), (**b**) monodehydroascorbate reductase (MDHAR), (**c**) dehydroascorbate reductase (DHAR), and (**d**) glutathione reductase (GR). Control, Tre, Cu, and Tre+Cu correspond to control, 10 mM trehalose, 100 μM CuSO_4_, and 10 mM trehalose + 100 μM CuSO_4_, respectively. Bars represent standard deviation (SD) of the mean (*n* = 3). Different letters indicate significant differences among treatments at *p* < 0.05, according to Duncan’s multiple range test.

The results related to the activities of the ascorbate-glutathione cycle enzymes are depicted in Fig. 4. Under Cu stress, APX activity increased by 96 and 82% at days 4 and 7, respectively, compared with control (Fig. 4a). APX activity did not increase at day 4, but increased by 23% at day 7 in the Tre-pretreated Cu-stressed seedlings compared with the Cu-stressed only seedlings. APX activity increased significantly in the Tre-pretreated seedlings at day 7 only, relative to control. MDHAR activity increased by 39 and 76% at days 4 and 7, respectively, in the Cu-stressed seedlings compared with control (Fig. 4b). On the other hand, MDHAR activity did not increase in the Tre-pretreated Cu-stressed seedlings compared with the Cu-stressed only seedlings, but the activity was still higher than that of control, by 15 and 57% at days 4 and 7, respectively. DHAR activity increased by 85 and 30% at days 4 and 7, respectively, in the Cu-stressed seedlings compared with control (Fig. 4c). On the other hand, DHAR activity did not increase at day 4, but increased by 39% at day 7 in the Tre-pretreated Cu-stressed seedlings compared with the Cu-stressed only seedlings. No significant change in DHAR activity was observed in the rice seedlings under non-stressed conditions upon Tre pretreatment over the experimental period. GR activity increased by 45 and 92% at days 4 and 7, respectively, in the Cu-stressed seedlings relative to control (Fig. 4d). In contrast, GR activity did not increase in the Tre-pretreated Cu-stressed seedlings compared with the Cu-stressed only seedlings, but it was 30 and 54% higher at days 4 and 7, respectively, than that of control.

### MG content and activity of Gly enzymes

**Figure 5:**
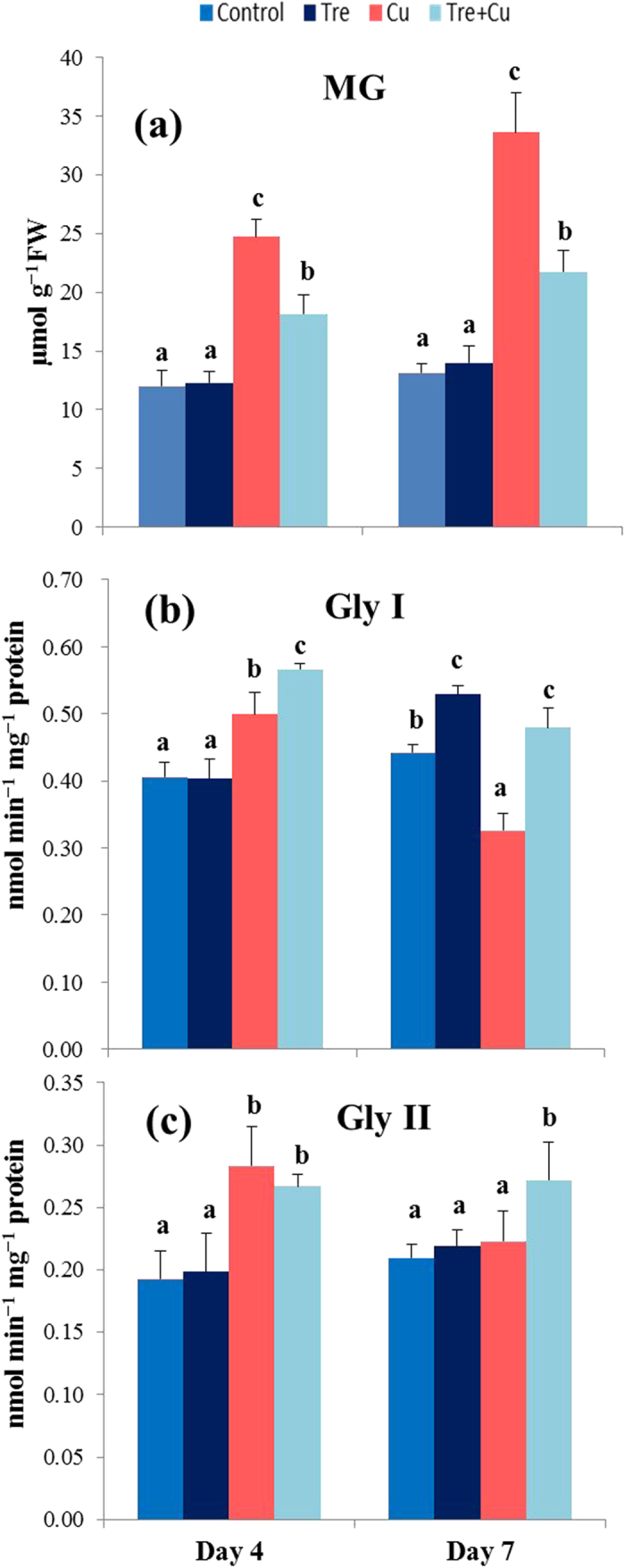
Effect of exogenous trehalose on activity of glyoxalase cycle enzymes in rice seedlings with or without Cu stress. (**a**) methylglyoxal (MG) content, (**b**) glyoxalase I (Gly I) activity, and (**c**) glyoxalase II (Gly II) activity. Control, Tre, Cu, and Tre+Cu correspond to control, 10 mM trehalose, 100 μM CuSO_4_, and 10 mM trehalose + 100 μM CuSO_4_, respectively. Bars represent standard deviation (SD) of the mean (*n* = 3). Different letters indicate significant differences among treatments at *p* < 0.05, according to Duncan’s multiple range test.

Under Cu stress, the MG level increased by 106 and 156% at days 4 and 7, respectively, compared with control (Fig. 5a). In comparison with the seedlings treated with Cu stress alone, the MG level decreased by 27 and 35% at days 4 and 7, respectively, in the seedlings treated with both Tre and Cu. Tre did not affect the level of MG in the Tre-pretreated seedlings under non-stressed conditions. On the other hand, the MG detoxifying enzymes Gly I and Gly II showed varying responses to Tre pretreatment in the presence or absence of Cu stress. Gly I activity increased by 22% at day 4 and decreased by 25% at day 7 in the Cu-stressed plants compared with control (Fig. 5b). Under Tre pretreatment, Gly I activity further increased in the Tre-pretreated Cu-stressed seedlings, making it higher than that of the Cu-stressed only seedlings by 14 and 45% at days 4 and 7, respectively. Under non-stressed conditions, Gly I activity increased by 20% in the Tre-pretreated seedlings at day 7 only relative to control. Gly II activity (Fig. 5c) increased 47% in the Cu-stressed seedlings over control at day 4, but the activity returned to the level in control at day 7. Conversely, Gly II activity did not increase significantly at day 4, but increased significantly at day 7 in the Tre-pretreated Cu-stressed seedlings compared with the Cu-stressed only seedlings. No significant change in Gly II activity was found under non-stressed conditions upon Tre pretreatment compared with control over the experimental period.

### Tre content

**Figure 6:**
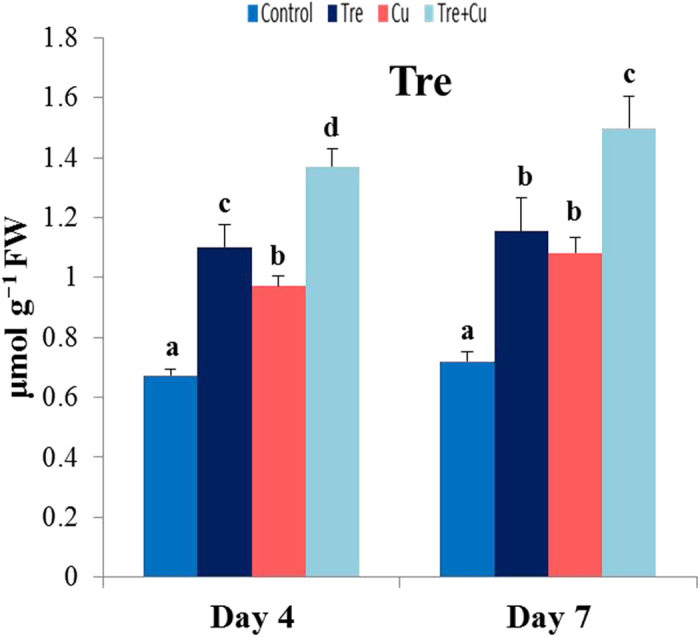
Effect of exogenous trehalose on endogenous trehalose content in rice seedlings with or without Cu stress. Control, Tre, Cu, and Tre+Cu correspond to control, 10 mM trehalose, 100 μM CuSO_4_, and 10 mM trehalose + 100 μM CuSO_4_, respectively. Bars represent standard deviation of the mean (*n* = 3). Different letters indicate significant differences among treatments at *p* < 0.05, according to Duncan’s multiple range test.

Tre level was estimated to observe the influence of exogenous Tre on the endogenous content of Tre and its contribution to the enhanced tolerance of the rice plants to Cu stress. Under Cu stress, the endogenous level of Tre increased in the stressed seedlings by 45 and 50% at days 4 and 7, respectively, relative to the unstressed control (Fig. 6). A further increase in the level of Tre was noted in the Cu-stressed seedlings due to Tre pretreatment, making the endogenous Tre level in the Tre-treated Cu-stressed seedlings significantly higher (41 and 39% at days 4 and 7, respectively) than that in the Cu-stressed only seedlings. Tre content also markedly increased (64 and 60% at days 4 and 7, respectively) in the Tre-pretreated seedlings compared with control.

## Discussion

In this study, we have provided an insight into how Tre regulates Cu homeostasis to provide protection to rice seedlings against excessive Cu. Prolonged Cu stress resulted in a decrease in the overall growth of rice seedlings (Table 1), perhaps by disturbing the functions of Cu-containing proteins and the cell metabolism of plants, which was attributed to Cu-induced phytotoxicity. However, Tre pretreatment enhanced the growth and biomass of Cu-stressed rice seedlings, demonstrating the ameliorating action of Tre in reducing Cu toxicity (Table 1), as also observed in maize under drought and salinity conditions^31^,^38^, which suggests the protecting role of Tre against a wide range of environmental stresses. Disturbance of water uptake and destruction of Chl, and thereby photosynthesis, have been regarded as characteristic symptoms of Cu stress^11^,^12^,^14^, as observed in this study (Table 1). More important, Tre-induced restoration of the levels of RWC and Chl in the Cu-stressed seedlings indicated an osmoprotective and membrane-protecting role of Tre for plants exposed to Cu stress. In addition, plants can respond rapidly to water imbalance by accumulating various osmolytes like Pro^39^. Many plants have been shown to accumulate Pro in large quantities when exposed to heavy metal stress^35^,^36^. However, despite its beneficial effects, Pro may be toxic if over-accumulated or applied in excessive concentrations^39^. In this work, we found that Cu stress induced a high increase in the Pro level, whereas Tre pretreatment reduced the accumulated Pro level in the Cu-stressed seedlings, which was still higher than that of control (Table 1). This result might indicate that Cu stress induced such a high increase in the Pro level that it caused metabolic perturbation, and that Tre pretreatment could provide osmotic protection to cells by adjusting the accumulated Pro to an optimal level.

In the current study, the degree of Cu toxicity was clearly seen with the levels of Cu uptake and accumulation in the roots and leaves upon extending the period of exposure to Cu, and thereby contributing to the reductions in plant growth and biomass. Our results indicated that Cu uptake and accumulation were much higher in the roots than in the leaves of the rice seedlings (Fig. 1), demonstrating that the roots are the primary site of Cu accumulation. Our results are in well agreement with the findings of Bouazizi *et al*.^4^ and Brackhage *et al*.^40^ who observed higher accumulation of Cu and arsenic in the roots of bean and wheat, respectively. Tre pretreatment significantly compromised Cu uptake and accumulation (Fig. 1), and was therefore useful in reducing Cu phytotoxicity. The restriction of Cu uptake and accumulation might have occurred because of the formation of a complex between Cu ions and Tre molecules, as observed between Tre and Cd when these were co-applied in a solution^41^. This pattern of Cu homeostasis might constitute an avoidance or exclusion strategy in reducing Cu accumulation and translocation in leaves, and thus allows higher Cu tolerance in rice seedlings.

In accordance with the higher accumulation of Cu, the present study demonstrated that increased levels of O_2_^•–^ and H_2_O_2_ (Table 2; Fig. 2) was associated with Cu-mediated oxidative burst in the rice seedlings. Cu-induced ROS levels also correlated with the substantial increase in MDA level (Table 2), which indicated severe membrane damage in the rice seedlings caused by Cu stress. In addition, increased LOX activity (Table 2) might have also contributed to oxidative damage by peroxiding membrane lipids. Tre treatment prior to Cu stress resulted in lower ROS production, as well as reduced LOX activity and MDA content, which might be achieved through Tre-mediated reduced Cu uptake (Fig. 1), direct ROS scavenging (Fig. 2), membrane stabilizing (Table 2), and modulation of the antioxidative mechanism involved in eliminating ROS (Figs 3,4). When the ROS level increases in plants exposed to stress, enhanced production of non-enzymatic antioxidants in plant cells, such as AsA and GSH, is crucial for minimizing ROS-induced oxidative stress^16^. The synchronized actions of these antioxidants drive the ascorbate-glutathione cycle to protect cells from ROS toxicity. Moreover, the reduced oxidized ratios of these antioxidants (AsA/DHA and GSH/GSSG) govern the redox balance of the cell^16^. The current study revealed that excessive Cu resulted in a decrease in the AsA level and AsA/DHA and GSH/GSSG ratios, but an increase in the GSH level (Table 3), which thereby impaired the ROS detoxification mechanism and consequently increased oxidative stress. Our results are supported by the work of Thounaojam *et al*.^14^ and Drazkiewicz *et al*.^15^, who investigated the effect of Cu stress on rice and *Arabidopsis thaliana*, respectively. On the other hand, Tre-induced amendment of the AsA level and redox status augmented the detoxification of ROS, which prevented oxidative damage in the Cu-exposed seedlings. Additionally, induced GSH synthesis was observed in this study in the rice plants treated with excessive Cu (Table 3), as also reported in other plant species under heavy metal stress^36^,^42^. However, Tre pretreatment resulted in a decreased GSH level in Cu-stressed seedlings (Table 3), which might be attributed to the Tre-induced reduction in Cu uptake and accumulation (Fig. 1). Furthermore, the increased activities of GSH-metabolizing enzymes (GPX, GST, DHAR, and Gly I) observed in the Cu-stressed plants upon Tre pretreatment might also curtail the cellular GSH level (Table 3; Figs 3c,d,4c,5b).

SOD, CAT, peroxidases, and ascorbate-glutathione cycle enzymes constitute the major enzymatic network that detoxifies ROS^15^. SOD, a frontline protective enzyme, converts O_2_^•−^ into H_2_O_2_, which is then subsequently removed by CAT and peroxidases. SOD and CAT activities in seedling leaves considered as biomarkers for growth and development under heavy metal stresses^43^. In this study, a Cu-induced increase in SOD activity did not correlate with the level of O_2_^•−^ (Figs 2a,3a), indicating that this level of SOD activity might not be sufficient to neutralize O_2_^•−^ toxicity. In addition, a profound decrease in CAT activity in the Cu-stressed seedlings trapped H_2_O_2_ and accelerated the accumulation of H_2_O_2_ (Table 2; Figs 2b,3b). As a consequence, excessive Cu might have amplified the Haber-Weiss reaction to produce deadly OH^•^. However, Tre pretreatment resulted in a further increase in SOD activity and countered the inhibition of CAT activity, particularly at day 7 of Cu exposure, which implied that Tre provided a safeguard against O_2_^•−^ and H_2_O_2,_ as well as OH^•^. A similar phenomenon was found in wheat under heat stress^30^, which supports our finding. Another important mechanism in regulating heavy metal-induced toxicity is associated with the GSH-dependent conjugation of lipid hydroperoxides and endobiotic substrates by GPX and GST^26^. Studies on hyperaccumulating and transgenic plants suggested that enhanced activities of GPX and GST conferred higher tolerance to abiotic stresses, including heavy metals^44^. In the present study, the enhanced activities of GPX and GST (Fig. 3c,d) due to Tre pretreatment suggested that there might be GSH-dependent peroxide scavenging that led to reduced oxidative damage. It might also be that the higher GST activity enhanced the membrane-protecting role of Tre by conjugating GSH with various Cu-induced electrophiles, which thereby prevented them from further damaging the membrane.

The ascorbate-glutathione cycle involves a series of reactions with four enzymes (APX, MDHAR, DHAR, and GR) that act in concert in H_2_O_2_ metabolism^16^,^26^. This cycle also appears to play a key role in maintaining cellular redox balance, especially under metal stress conditions^25^,^45^. However, in this study, despite the stimulation of the activities of all four enzymes upon Cu stress, the H_2_O_2_ level remained significantly higher (Table 2; Fig. 4a–d), which indicated that the amount of stimulation was not up to the requisite level in lowering excess H_2_O_2_. In another study, Thounaojam *et al*.^14^ observed an accumulation of H_2_O_2_ even after increasing the activity of H_2_O_2_-scavenging enzymes, suggesting that production of H_2_O_2_ exceeded ROS scavenging capacity. In contrast, Tre, by intensifying APX and DHAR activities as well as keeping MDHAR and GR activities above the control level, coordinates the activities of these four enzymes with enhanced CAT and GPX activities, and thereby contributes well to regulating the H_2_O_2_ level (Table 2; Figs 2, 3, 4).

Under abiotic stress conditions, MG is overproduced, which in turn contributes to cellular toxicity as well as oxidative damage in plant tissues^18^,^19^,^21^. Plant cells detoxify MG by using the Gly system^21^. In the present study, a time-dependent increase in the MG level correlated with the decreased activities of Gly I and Gly II (Fig. 5), and thereby intensified Cu toxicity in the rice seedlings. In contrast, Tre pretreatment detoxified MG by up-regulating the Gly I and Gly II activities, which indicated a positive effect of Tre in amplifying MG detoxification under Cu stress. Several reports have suggested that the antioxidant and Gly systems are closely linked and they act simultaneously in conferring tolerance against multiple stresses, including heavy metals^17^,^23^,^24^,^25^. It has also been reported that the enhanced activities of Gly enzymes and antioxidant enzymes (APX, GST, and GPX) provided higher tolerance to Zn toxicity in *Brassica oleracea*^46^. Our study suggested that Tre might enhance rice tolerance to Cu stress by coordinating the actions of the antioxidant and Gly systems to mitigate Cu-induced ROS and MG toxicity.

Although Tre accumulates in negligible amounts in crop plants, it has been considered a quantitatively important compatible solute and stress protectant^29^. Non-stressed and stressed rice seedlings pretreated with Tre increased the endogenous level of Tre (Fig. 6), which indicated that Tre was readily absorbed by the roots and easily transported to the aerial parts. Our results corroborate previous findings^28^,^30^,^31^,^32^,^33^,^34^ and suggest that external application of Tre could be an alternative approach to modify the level of endogenous Tre, and thus potentially strengthen plant capacity to withstand the deleterious effects of environmental threats. From this point of view, we assume that additional protection against Cu toxicity in the rice seedlings was attributed to the increase in the endogenous level of Tre.

In conclusion, prolonged exposure to excessive Cu resulted in serious toxic effects on the rice seedlings by affecting their physiological and biochemical attributes. In contrast, Tre pretreatment has been shown to be beneficial in alleviating Cu toxicity, which was mainly attributed to the ability of Tre (i) to restrict Cu uptake and accumulation to maintain Cu homeostasis, and (ii) to induce production of antioxidant and Gly enzymes to alleviate excessive Cu-triggered oxidative stress. Current data suggests that enhancing the level of endogenous Tre by supplementation or genetic engineering could be used as an important strategy in mitigating Cu toxicity, and perhaps other heavy metal toxicity, in rice or other crop plants.

## Methods

### Plant materials, growth conditions, and treatments

Rice (*Oryza sativa* L. cv. BRRI dhan29) seeds were surface sterilized with 1% (v/v) sodium hypochlorite solution for 20 min, washed with distilled water, and imbibed for 24 h. The seeds were sown on plastic nets floating on distilled water in 250 mL plastic beakers and kept in the dark at 28 ± 2 °C for germination. After 48 h, uniformly germinated seeds were transferred to a growth chamber and grown in a commercial hydroponic solution (Hyponex, Japan) diluted according to the manufacturer’s instructions. The nutrient solution consisted of 8% N, 6.43% P, 20.94% K, 11.8% Ca, 8% S, 3.08% Mg, 0.07% B, 0.24% Fe, 0.03% Mn, 0.0014% Mo, 0.008% Zn, and 0.003% Cu. The seedlings were grown under controlled conditions (photon density: 100 μmol m^−2^ s^−1^, temperature: 26 ± 2 °C, RH: 65–70%). Each plastic beaker contained about 60 rice seedlings. The nutrient solution (pH 5.5) was renewed every four days. At day 10 after sowing, seedlings were pretreated with 10 mM Tre in the hyponex solution for 48 h. Tre-pretreated and non-pretreated rice seedlings were then subjected to 100 μM CuSO_4_ in the hyponex solution. This CuSO_4_ concentration was selected based on our previous experiments^12^,^35^,^47^. Based on literature^33^,^34^,^48^ and our preliminary experiments with a range of Tre concentrations (5, 10, 15, and 20 mM), we observed that 10 mM Tre was optimally effective in alleviating Cu-induced toxic symptoms. Therefore, our experiment consisted of 4 treatments as follows: (i) control, (ii) 10 mM trehalose (Tre), (iii) 100 μM CuSO_4_ (Cu), and (iv) 10 mM trehalose + 100 μM CuSO_4_ (Tre+Cu). The second leaf of rice seedlings was harvested at day 4 and 7 after Cu-stress treatment to determine various physiological and biochemical parameters. Each treatment was replicated three times under the same experimental conditions.

### Determination of plant height, FW, DW, total Chl, RWC, and Pro content

Just after harvest, plant height was determined by measuring the length from the bottom of the main stem to the end of the emerging third leaf. To determine FW, seedlings were separated from the culture medium and the roots were washed thoroughly with distilled water followed by blotting with tissue paper. After separating adherent seeds, 10 seedlings from each treatment were weighed to determine FW (g seedling^−1^). To quantify DW, 10 seedlings from each treatment were oven dried at 80 °C for 48 h and expressed as g seedling^−1^. To estimate total Chl content, leaves (0.5 g) were extracted in 80% chilled acetone and Chl was estimated according to the formula of Arnon^49^.

Total Chl content (mg g^−1^ FW) = (20.2 × D645 + 8.02 × D663) × V/(1000 × W); where V = volume of 80% (v/v) acetone (mL), W = weight of sample (g).

RWC was determined as described by Mostofa *et al*.^12^. Pro content was determined according to the method of Bates *et al*.^50^ with minor modifications. Fresh leaf samples (0.5 g) were homogenized with 5 mL of 3% aqueous sulfosalicylic acid and the homogenate was centrifuged at 11,500 × g for 15 min. Supernatant (2 mL) was mixed with 2 mL of glacial acetic acid and 2 mL of acid ninhydrin solution. The resultant mixture was boiled at 100 °C for 1 h and then transferred to ice to stop the reaction. The developed red color was extracted with 4 mL toluene and absorption of the chromophore was measured at 520 nm. Pro concentration was calculated using a calibration curve developed with Pro standard.

### Measurement of Cu content and its accumulation in roots and leaves

To determine Cu content, the root and leaf samples were harvested separately and the roots were washed thoroughly with distilled water to remove Cu ions adhering to the root surface. The root and leaf samples were oven dried at 80 °C for 48 h. Dried samples (0.1 g) were ground and digested with a HNO_3_:HClO_4_ (5:1 v/v) mixture at 80 °C until the yellow color disappeared. The Cu content in the roots and leaves was measured by using flame atomic absorption spectrophotometry (Z-5000; Hitachi, Japan). The concentration and accumulation of Cu in the roots and leaves were determined by the following formula:

Cu concentration (mg g^−1^ DW) = reading of AAS × dilution factor/dry wt. of roots or leaves

Cu accumulation (mg seedling^−1^) = conc. of Cu × dry wt. of roots or leaves of each seedling.

### Determination of lipid peroxidation and H_2_O_2_ content

Lipid peroxidation of the second leaves was measured by estimating MDA according to the method of Heath and Packer^51^ using an extinction co-efficient of 155 mM^−1^ cm^−1^. H_2_O_2_ was extracted and determined after reaction with 0.1% TiCl_4_ in 20% H_2_SO_4_ following the method of Yu *et al*.^52^.

### Histochemical detections of ROS (O_2_^.–^ and H_2_O_2_)

O_2_^.–^ and H_2_O_2_ were detected in rice leaves according to the method of Mostofa and Fujita^12^. In brief, the second leaves were stained in 0.1% nitroblue tetrazolium (NBT) solution or 1% 3,3’-diaminobenzidine (DAB) solution to detect O_2_^.–^ and H_2_O_2_, respectively. After 24 h of incubation, leaves were decolorized by immersing them in boiling ethanol to detect the blue insoluble formazan (for O_2_^.–^) or deep brown polymerization product (for H_2_O_2_). After cooling, photographs were taken by placing the leaves between two glass plates.

### Estimation of non-enzymatic antioxidants

Fresh leaves (0.5 g) were homogenized in 3 mL of ice-cold 5% meta-phosphoric acid containing 1 mM EDTA and centrifuged at 11,500 × g for 15 min. Reduced and total AsA content were determined following the method of Dutilleul *et al*.^53^ with minor modifications. To estimate the total AsA level, the oxidized fraction was reduced by 0.1 M dithiothreitol. Reduced and total AsA content were assayed at 265 nm in 100 mM K-phosphate buffer (pH 7.0) with 1.0 U of ascorbate oxidase (AO). DHA content was calculated by deducting the reduced AsA amount from total AsA content. Based on enzymatic recycling, reduced GSH, GSSG, and total glutathione (GSH + GSSG) content were determined according to the method of Griffiths^54^. GSSG content was determined after removing GSH by 2-vinylpyridine derivatization. GSH concentration was measured after subtracting the value of GSSG from the total GSH level.

### Extraction and assay of enzymes

To extract enzymes, fresh leaf samples (0.5 g) were homogenized separately with a reaction mixture containing 50 mM K-phosphate buffer (pH 7.0), 100 mM KCl, 1 mM AsA, 5 mM *β*-mercaptoethanol, and 10% (w/v) glycerol in pre-chilled mortars. The homogenate was centrifuged at 11,500 × g for 15 min and the resultant supernatants were collected to analyze enzyme activities and protein content. All procedures were performed at 0–4 °C.

LOX (EC 1.13.11.12) activity was estimated according to the method of Doderer *et al*.^55^ by monitoring the increase in absorbance at 234 nm using linoleic acid as a substrate. SOD (EC 1.15.1.1) activity was estimated according to the method of El-Shabrawi *et al*.^24^, which is based on a xanthine-xanthine oxidase system. The reaction mixture contained K-phosphate buffer (50 mM), NBT (2.24 mM), CAT (0.1 units), xanthine oxidase (0.1 units), xanthine (2.36 mM), and enzyme extract. SOD activity was expressed as units (that is, the amount of enzyme required to inhibit NBT reduction by 50%) min^−1^ mg^−1^ protein. CAT (EC 1.11.1.6) activity was measured according to the method of Mostofa *et al*.^56^. APX (EC 1.11.1.11) activity was determined by monitoring the decrease in absorbance at 290 nm as AsA was oxidized, according to the method of Nakano and Asada^57^. MDHAR (EC 1.6.5.4) activity was measured by using 1 U of AO and the oxidation rate of NADPH was determined at 340 nm^58^. DHAR (EC 1.8.5.1) activity was measured by monitoring the formation of AsA from DHA at 265 nm using GSH^57^. GR (EC 1.6.4.2) activity was quantified by monitoring the decrease in the absorbance of NADPH at 340 nm for GSSG-dependent oxidation of NADPH, as described by Foyer and Halliwell^59^. GST (EC 2.5.1.18) activity was determined using the method of Mostofa *et al*.^11^. GPX (EC: 1.11.1.9) activity was measured as described by Mostofa *et al*.^56^ using H_2_O_2_ as a substrate. Gly I (EC 4.4.1.5) assay was carried out according to the method of Hossain *et al*.^18^ using an extinction co-efficient of 3.37 mM^−1^ cm^−1^. Gly II (EC 3.1.2.6) activity was determined according to the method of Mostofa *et al*.^56^ using an extinction co-efficient of 13.6 mM^−1^ cm^−1^. Total protein content in the extracts was estimated with the dye-binding method of Bradford^60^ using bovine serum albumin (BSA) as standard.

### Determination of Tre content

Tre content in the second leaves was determined following the method described by Li *et al*.^61^ with minor modifications. The leaves (1.0 g) were homogenized in 5 mL of 80% (v/v) hot ethanol and centrifuged at 11,500 × g for 20 min. The supernatants were dried at 80 °C followed by re-suspension in 5 mL distilled water. The solution (100 μL) was mixed with 150 μL 0.2 N H_2_SO_4_ and boiled at 100 °C for 10 min to hydrolyze any sucrose or glucose-1-phosphate, then chilled on ice. NaOH (0.6 N, 150 μL) was added to the above mixture and boiled for 10 min to destroy reducing sugars, then chilled again. Anthrone reagent (2.0 mL; 0.2 g anthrone per 100 mL of 95% H_2_SO_4_) was added to the above mixture and boiled for 10 min to develop a color and then chilled again. The absorbance was recorded at 630 nm and Tre concentration was calculated as μmol g^−1^ FW using a standard curve developed with commercial Tre.

### Determination of MG content

MG content was measured following the method described by Wild *et al*.^62^ with modifications. Leaves (0.25 g) were homogenized in 2.5 mL 0.5 M perchloric acid and incubated for 15 min on ice. The extract was centrifuged for 10 min at 11,200 × g at 4 °C and 1 mL supernatant was transferred to a fresh microcentrifuge tube. Charcoal (10 mg mL^−1^) was added and kept at room temperature for 15 min to decolorize the supernatant. The mixture was centrifuged for 10 min at 11,000 × g and the supernatant was neutralized by saturated K_2_CO_3_. The extract was kept at room temperature for 15 min and centrifuged at 11,200 × g for 10 min. In a total volume of 1 mL, 650 μL of neutralized supernatant, 330 μL of 100 mM sodium dihydrogen phosphate buffer (pH 7.0), and 20 μL of freshly prepared 0.5 M N-acetyl-L-cysteine were added and incubated for 15 min. Formation of N-α-acetyl-S-(1-hydroxy-2-oxo-prop-1-yl) cysteine was recorded at a wavelength of 288 nm and MG content was calculated by using a standard curve of known concentration of MG.

### Statistical analysis

The data were subjected to one-way analysis of variance (ANOVA) and different letters indicate significant differences between treatments at *p* < 0.05, according to Duncan’s multiple range test (DMRT) using IRRISTAT version 3 (International Rice Research Institute, Biometrics Unit, Manila, Philippines). Data represented in the Tables and Figures are means ± standard deviations (SD) of three replicates for each treatment.

## Additional Information

**How to cite this article**: Mostofa, M. G. *et al*. Physiological and biochemical mechanisms associated with trehalose-induced copper-stress tolerance in rice. *Sci. Rep*. **5**, 11433; doi: 10.1038/srep11433 (2015).

## Figures and Tables

**Table 1:** Effect of exogenous trehalose on plant growth, relative water content (RWC), total chlorophyll (Chl) content, and proline (Pro) content in rice seedlings with or without Cu stress.

Duration	Treatment	Plant height (cm seedling^−1^)	FW (g seedling^−1^)	DW (g seedling^−1^)	Leaf RWC (%)	Total Chl (mg g^−1^ FW)	Pro (μmol g^−1^ FW)
Day 4	Control	13.57 ± 0.60^b^	0.124 ± 0.003^c^	0.029 ± 0.001^b^	98.78 ± 0.19^c^	1.50 ± 0.05^b^	0.28 ± 0.07^a^
	Tre	13.07 ± 0.46^b^	0.129 ± 0.004^c^	0.031 ± 0.001^b^	98.34 ± 0.90^c^	1.50 ± 0.02^b^	0.25 ± 0.12^a^
	Cu	11.80 ± 0.62^a^	0.083 ± 0.003^a^	0.024 ± 0.001^a^	81.41 ± 2.14^a^	1.00 ± 0.08^a^	1.65 ± 0.11^c^
	Tre+Cu	13.07 ± 0.38^b^	0.105 ± 0.006^b^	0.029 ± 0.001^b^	93.57 ± 2.12^b^	1.46 ± 0.01^a^	0.89 ± 0.08^b^
Day 7	Control	14.40 ± 0.85^b^	0.180 ± 0.006^c^	0.036 ± 0.001^c^	98.44 ± 1.40^c^	1.53 ± 0.05^c^	0.35 ± 0.04^a^
	Tre	14.23 ± 0.40^b^	0.180 ± 0.007^c^	0.036 ± 0.001^c^	98.55 ± 0.83^c^	1.49 ± 0.04^c^	0.29 ± 0.06^a^
	Cu	11.77 ± 0.76^a^	0.067 ± 0.006^a^	0.022 ± 0.001^a^	67.05 ± 2.19^a^	0.60 ± 0.06^a^	2.33 ± 0.25^c^
	Tre+Cu	14.00 ± 0.46^b^	0.117 ± 0.006^b^	0.027 ± 0.001^b^	79.01 ± 2.03^b^	0.86 ± 0.05^b^	1.11 ± 0.13^b^

Control, Tre, Cu, and Tre+Cu correspond to control, 10 mM trehalose, 100 μM CuSO_4_, and 10 mM trehalose + 100 μM CuSO_4_, respectively. FW, fresh weight; DW, dry weight. Values are means ± SD of three independent replications (n = 3). Different letters within the column indicate statistically significant differences between treatments, according to Duncan’s multiple range test (*p* < 0.05).

**Table 2:** Effect of exogenous trehalose on levels of malondialdehyde (MDA) and hydrogen peroxide (H_2_O_2_), and lipoxygenase (LOX) activity in rice seedlings with or without Cu stress.

Duration	Treatment	H_2_O_2_ (nmol g^−1^ FW)	MDA (nmol g^−1^ FW)	LOX (nmol min^−1^ mg^−1^ protein
Day 4	Control	14.43 ± 1.65^a^	16.19 ± 1.45^b^	10.24 ± 1.47^a^
	Tre	15.41 ± 1.25^a^	14.38 ± 0.56^a^	12.95 ± 1.64^b^
	Cu	24.06 ± 1.44^b^	35.79 ± 2.74^d^	16.69 ± 1.34^d^
	Tre+Cu	22.88 ± 2.39^b^	29.69 ± 1.60^c^	14.72 ± 1.53^c^
Day 7	Control	16.72 ± 0.98^a^	17.39 ± 1.90^a^	14.67 ± 1.28^a^
	Tre	18.23 ± 1.83^a^	16.61 ± 1.32^a^	15.41 ± 1.68^a^
	Cu	36.58 ± 2.18^c^	43.72 ± 1.74^c^	23.63 ± 1.70^c^
	Tre+Cu	20.96 ± 1.53^b^	30.69 ± 1.78^b^	17.92 ± 1.90^b^

Control, Tre, Cu, and Tre+Cu correspond to control, 10 mM trehalose, 100 μM CuSO_4_, and 10 mM trehalose + 100 μM CuSO_4_, respectively. FW, fresh weight. Values are means ± SD of three independent replications (n = 3). Different letters within the column indicate statistically significant differences between treatments, according to Duncan’s multiple range test (*p* < 0.05).

**Table 3:** Effect of exogenous trehalose on the levels of non-enzymatic antioxidants (ascorbic acid, AsA and glutathione, GSH) and their redox ratios (AsA/DHA, GSH/GSSG) in rice seedlings with or without Cu stress.

Duration	Treatment	Total AsA (nmol g^−1^ FW)	AsA/DHA ratio	GSH (nmol g^−1^ FW)	GSH/GSSG ratio
Day 4	Control	3979.25 ± 68.80^c^	3.68 ± 0.51^b^	492.56 ± 27.33^a^	31.04 ± 3.71^c^
	Tre	4133.52 ± 199.36^c^	4.83 ± 0.26^c^	462.74 ± 32.20^a^	32.04 ± 3.07^c^
	Cu	2412.68 ± 215.14^a^	1.21 ± 0.24^a^	815.54 ± 69.93^c^	17.38 ± 1.26^a^
	Tre+Cu	3344.98 ± 294.46^b^	3.16 ± 0.47^b^	767.92 ± 57.03^b^	20.07 ± 0.56^b^
Day 7	Control	4264.65 ± 213.20^c^	4.86 ± 0.63^c^	573.38 ± 41.18^a^	33.22 ± 0.15^c^
	Tre	4401.74 ± 139.84^c^	6.03 ± 0.64^d^	580.56 ± 46.11^a^	37.59 ± 4.43^d^
	Cu	1981.15 ± 159.26^a^	0.97 ± 0.18^a^	992.46 ± 20.12^c^	12.45 ± 2.31^a^
	Tre+Cu	3829.16 ± 150.62^b^	3.31 ± 0.31^b^	632.80 ± 53.85^b^	15.35 ± 1.07^b^

Control, Tre, Cu, and Tre+Cu correspond to control, 10 mM trehalose, 100 μM CuSO_4_, and 10 mM trehalose + 100 μM CuSO_4_, respectively. AsA, ascorbic acid; DHA, dehydroascorbic acid (oxidized ascorbic acid); FW, fresh weight; GSH, reduced glutathione; GSSG, glutathione disulfide (oxidized glutathione).Values are means ± SD of three independent replications (n = 3). Different letters within the column indicate statistically significant differences between treatments, according to Duncan’s multiple range test (*p* < 0.05).
